# Supplementary material for: Association of NUDT15 c.415C>T and FPGS 2572C>T Variants with the Risk of Early Hematologic Toxicity During 6-MP and Low-Dose Methotrexate-Based Maintenance Therapy in Indian Patients with Acute Lymphoblastic Leukemia
Source: Genes (Basel). 2020 May 28;11(6):594. doi: 10.3390/genes11060594 (PMC7349017; doi:10.3390/genes11060594)
Supplement: Supplementary file 1 [file genes-11-00594-s001.pdf]

Table S1: MCP 841 PROTOCOL (I 2 A PROTOCOL)

Phase	Route of Administration	Chemotherapy Drug	Dose	Administered on days
**Induction (I1)**	Intravenous (IV)	Vincristine	1.4 mg/m^2	1,8,15,22 and 29
	Intravenous (IV)	Daunorubicin	30 mg/m^2	8,15 and 29
	Subcutaneous (SC)	L-Asparaginase	60000 IU/m^2	2-20 alternate day
	Per Oral (PO)	Prednisolone	40 mg/m^2	1-28
	Intra Thecal (IT)	Methotrexate	12 mg/m^2	8, 15 and 22
**Induction 2 (I2)**	Per Oral (PO)	6-Mercaptopurine	75 mg/m^2	Daily 1-7 and 15-21
	Intravenous (IV)	Cyclophosphamide	750 mg/m^2	1 and 15
	Intra Thecal (IT)	Methotrexate	12 mg/m^2	1, 8, 15 and 22
		Cranial Irradiation	2000 cGy	10 days
**Repeat Induction -1 (RI1)**				
**Consolidation (C)**	Intravenous (IV)	Cyclophosphamide	750 mg/m^2	1 and 15
	Intravenous (IV)	Vincristine	1..4mg/m^2	1 and 15
	Subcutaneous (SC)	Cytarabine	70 mg/m^2 every 12 hrs × 6 doses	1-3 and 15-17
	Per Oral (PO)	6-Mercaptopurine	75 mg/m^2	1-7 and 15-21
	Per Oral (PO)	Prednisone	40 mg/m^2	1-7
**Maintenance**	Intravenous (IV)	Vincristine	1.4 mg/m^2	On day 1
	Intravenous (IV)	Daunorubicin	30 mg/m^2	On day 1
	Subcutaneous (SC)	L-Asparaginase	60000 IU/m^2	1,3,5 and 7
	Per Oral (PO)	Methotrexate	15 mg/m^2	Once a week, missing every 4th week for a total of 12 weeks. Begin on day 15
	Per Oral (PO)	6-Mercaptopurine	75 mg/m^2	Daily 3 weeks out of every 4 for a total of 12 weeks. Begin on day 15

Table S2: ADULT ALL PROTOCOL (G-MALL)

Phase	Route of Administration	Chemotherapy Drug	Dose	Administered on days
**Induction (I1) (0-4 Weeks)**	Per Oral (PO)	Prednisolone	60 mg/m^2	1- 28
	Subcutaneous (SC)	L-Asparaginase	5000 U/m^2	2,4,6,8,10,12,14
	Intravenous (IV)	Vincristine	1.5 mg/m^2	1,8,15 and 22
	Intravenous (IV)	Daunorubicin	25 mg/m^2	1,8,15 and 22
**Induction 2 (I2) 5-8 Weeks**	Intravenous (IV)	Cyclophosphamide	650 mg/m^2	1, 15 ,29
	IV infusion (over 1 hr)	Ara -C	75 mg/m^2	1-4, 8-11, 15-18, 22-25
	Per Oral (PO)	6-Mercaptopurine	60 mg/m^2	1 - 28
	Intra Thecal (IT)	Methotrexate	4 Doses	1, 8, 15 and 22
**Interim phase(2 phases)**	IV infusion (over 1 hr)	Ara-C	75mg/m^2	1-5
	IV infusion (over 1 hr)	Etoposide	50mg/m^2	1-5
**Cranial RT (Sandwich between the two phases)**		CNS 24 Gy Radiation		
**Consolidation (Phase I: 4 Weeks)**	Per Oral (PO)	Dexamethasone	10 mg/m^2	1-28
	Intravenous (IV)	Vincristine	1.5mg/m^2	1-28
	Intravenous (IV)	ADR	25 mg/m^2	1, 8, 15 and 22
**Consolidation (Phase II: 2 Weeks)**	Intravenous (IV)	Chlorotoxin (CTX)	650 mg/m^2	Day 2
	Subcutaneous (SC)	Ara-C	75 mg/m^2	4-7, 11-14
	Per Oral (PO)	6-Mercaptopurine	75 mg/m^2	1-14
**Re-intensification (3 Phases Each)**	IV Infusion (over 1 hr)	Ara-C	75mg/m2	Daily for 5 days
	IV Infusion (over 1 hr)	Etoposide	50 mg/m^2	Daily for 5 days
**Maintenance (24 months)**	Per Oral (PO)	Methotrexate	20 mg/m^2	Weekly
	Per Oral (PO)	6-Mercaptopurine	50 mg/m^2	Daily

Table S4. Association of methotrexate polyglutamates with hematological toxicity in the study group			
MTXPGs (pmol/mg/8X10^{12} RBCs)	Severe (grade 3-4)<br>N; Median (Q1 to Q3)	Mild (grade 0-2)<br>N; Median (Q1 to Q3)	p-value
Anaemia			
PG3	16; 7.28 (4.96 to 9.53)	38; 6.75 (3.21 to 10.24)	0.71
PG4	12; 17.36(7.16 to 33.67)	33; 13.89(7.36 to 28.63)	0.79
PG5	6; 4.31 (3.38 to 6.79)	12; 3.94(2.14 to 6.01)	0.64
Leukopenia			
PG3	24; 7.92(5.57 to 15.96)	30; 5.94(3.10 to 8.60)	0.07
PG4	21; 17.44(5.91 to 39.31)	24; 13.63(7.73 to 27.42)	0.42
PG5	9; 4.48(3.12 to 5.97)	10; 3.62(1.72 to 4.48)	0.19
Thrombocytopenia			
PG3	17; 7.42(5.15 to 9.47)	37; 6.71(3.43 to 12.57)	0.74
PG4	14; 19.75(11.87 to 28.09)	31; 13.38(7.01 to 28.70)	0.62
PG5	9; 4.16 (3.39 to 4.90)	10; 3.62(1.40 to 5.93)	0.36
N indicates a number of patients in that particular group; MTXPGs-Methotrexate polyglutamates. IQ, inter-quartile range; NA-not applicable; Mann-Whitney U-test was used.			

Table S3. Methotrexate polyglutamate levels between patients with and without relapse			
MTXPGs (pmol/mg/8X10^{12} RBCs)	Relapse	No relapse	p-value
	N; Median(IQ)	N; Median(IQ)	
PG3	17; 5.68(2.94 to 9.46)	38; 7.52(4.76 to 10.07)	0.27
PG4	13; 21.41(4.60, 29.02)	13; 15.28(7.75, 28.6)	0.97
PG5	6; 3.54(1.72, 4.57)	12; 4.16(3.58, 6.04)	0.49
N, the number of patients in that particular group; MTXPGs-Methotrexate polyglutamates. IQ, inter-quartile range; Mann-Whitney U-test was used.			

Table S5 . Comparison of MTXPG3-5 levels across various genotypes

Genetic variants	PG3	PG4	PG5
	**Median (IQ); [n]**	**Median (IQ); [n]**	**Median (IQ); [n]**
**Variants in MTX transporter genes**			
RFC rs1051266			
AA	5 (3-10); [11]	27 (12-30); [9]	5.9 (3.65-24.70); [5]
AG	7 (5-10); [43]	14.5 (6.25-28.50); [36]	3.90 (2.40-5.12); [14]
**p-value**	0.31	0.16	0.22
SLCO1B1 rs4149056			
TT	7 (4.75-10.25); [50]	16 (8.0-29.0); [42]	4.30 (3.05-6.05); [17]
TC	5 (1.25-8.75); [4]	27 (1-34); [3]	3.40 (2.32-11.28); [2]
**P-value**	0.20	0.96	0.35
MDR rs1128503			
CC	7 (4-11); [7]	13 (3-31); [7]	5.9 (2.70-28.10); [5]
CT	8 (4.50-13.75); [20]	12.50 (7.75-24.50); [18]	4.30 (3.50-10.40); [3]
TT	6.0 (4.0-10.0); [27]	22.0 (14.74-32.75); [20]	3.70 (2.60-4.80); [11]
**P-value**	0.85	0.27	0.40
*MDR*rs1045642			
CC	5 (3.00-10.00); [7]	13 (4.00-31.00); [7]	5.90 (1.20-43.40); [3]
CT	8 (6.00-13.00); [21]	13 (8-29); [19]	4.25 (3.95-7.10); [6]
TT	7 (3.75-9.25); [26]	21 (6-34); [19]	3.65 (2.40-5.12); [10]
**P-value**	0.53	0.85	0.62
MDR rs2032582			
GG	7 (2.50-8.50); [5]	8 (5.50-27.0); [5]	12.80 (1.20-43.40); [3]
GT+GA	8 (3.00-16.50); [18]	12.50 (7.25-28.50); [16]	4.55 (4.02-7.02); [6]
TT+TA	7 (5.00-10.00); [31]	21(13-32.75); [24]	3.65 (2.40-4.88); [10]
**P-value**	0.62	0.46	0.29
**Variants in genes encoding MTX metabolizing enzymes**			
FPGS rs10106			
TT	6 (3-10); [15]	14 (6.5-31.50); [13]	4.35 (2.40-5.70); [4]
TC	7 (4-15); [27]	21 (7-31); [23]	13 (9-25); [11]
CC	8 (6-9); [12]	13 (9-25.5); [9]	3.8 (2.82-10.6); [4]
**P-value**	0.76	0.81	0.95
FPGS rs1544105			
CC	6 (3-10); [15]	14 (6.50-31.50); [13]	4.35 (2.40-5.70); [4]
CT	7 (4-13.75); [28]	19 (6.25-30.50); [24]	4.55 (3.22-9.30); [12]
TT	8 (6-9); [11]	13.50 (10.75-27.25); [8]	3.50 (2.6-4.10); [3]
**P-value**	0.75	0.94	0.46
GGH rs3758149			
CC	8 (5-9); 22	13 (5.50-28.50); 20	3.60 (2.45-4.65); 9
CT	7 (4-11); 27	21 (10.50-30.00); 21	5.10 (3.82-6.07); 8
TT	6 (2.50-17.50); 5	8.50 (1.50-35.0); 4	7.70 (1.95-13.10); 2
**P-value**	0.70	0.32	0.37
GGH rs11545078			
CC	7 (4-9); 39	13 (6.50to 28); 33	4.20 (3.30-5.95); 13
CT+TT	8 (6-15); 15	22 (14.75-37.75); 12	4 (2.32-7.77); 6
**P-value**	0.38	0.26	0.93
n, the number of patients in that particular group; MTXPGs-Methotrexate polyglutamates. IQ, inter-quartile range; Mann-Whitney U-test or Kruskal-Wallis test was used.			

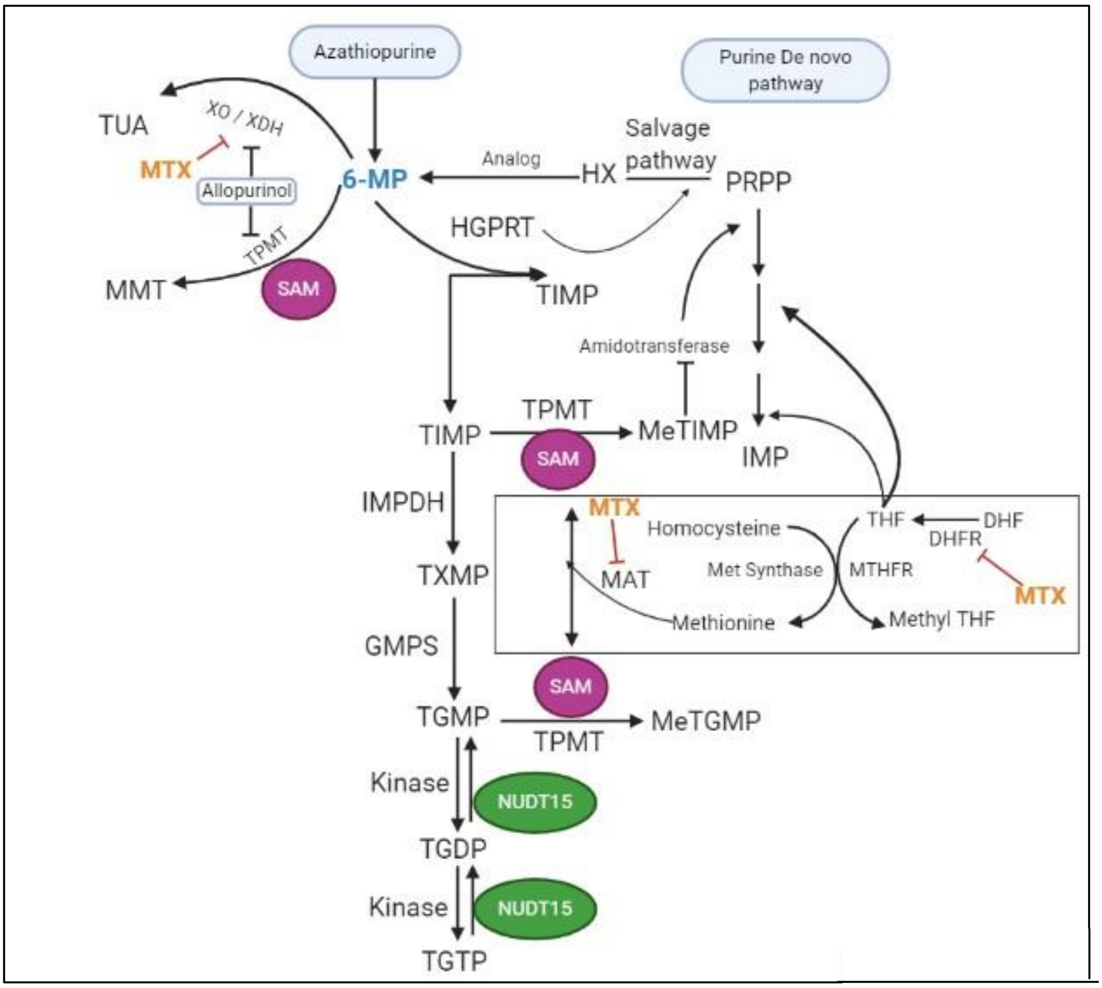

Figure S1: Synergetic effects of 6-mercaptopurine (6-MP) and methotrexate (MTX). TUA: Thiouric acid; MMT: methyl-mercaptopurine; XO/XDH: xanthine oxidase/xanthine dehydrogenase; TPMT: thiopurine methyltransferase; HGPRT: hypoxanthine guanine phosphoribosyl transferase; HX: hypoxanthine; PRPP: Phosphoribosyl pyrophosphate; IMP: inosine monophosphate; TIMP: thioinosine monophosphate; MeTIMP: methyl- thio-inosine monophosphate; IMPDH: inosine monophosphate dehydrogenase; TXMP: thioxanthosine monophosphate; GMPS: guanosine monophosphate synthetase; TGMP: thioguanosine monophosphate; MeTGMP: methyl-thioguanosine monophosphate; SAM: S-adenosyl-methionine; THF: tetrahydrofolate, DHF: dihydrofolate; Met synthase: methionine synthase; MTHFR: methyl tetrahydrofolate reductase; TGDP: thioguanosine diphosphate; TGTP: thioguanosine triphosphate; NUDT15: nucleoside diphophate–linked moiety X-type motif 15.

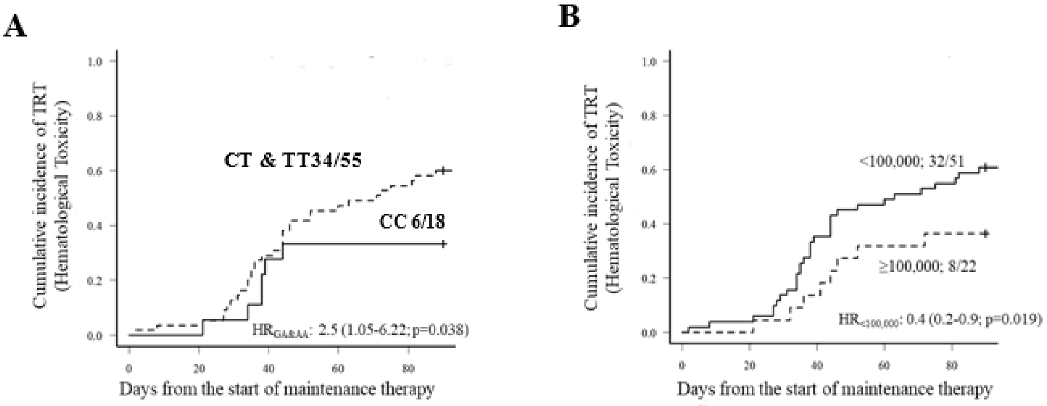

Figure S2: Association early hematological TRT during maintenance therapy in ALL patients with that of (A) *FPGS* rs1544105 variant (n=73); (B) platelet count at the time of diagnosis. The number of events / total numbers in each group, p values and hazards ratios for cumulative incidences of severe hematological toxicity are presented on the plots. *FPGS* variant association with TRT is not significant after multiple testing correction.

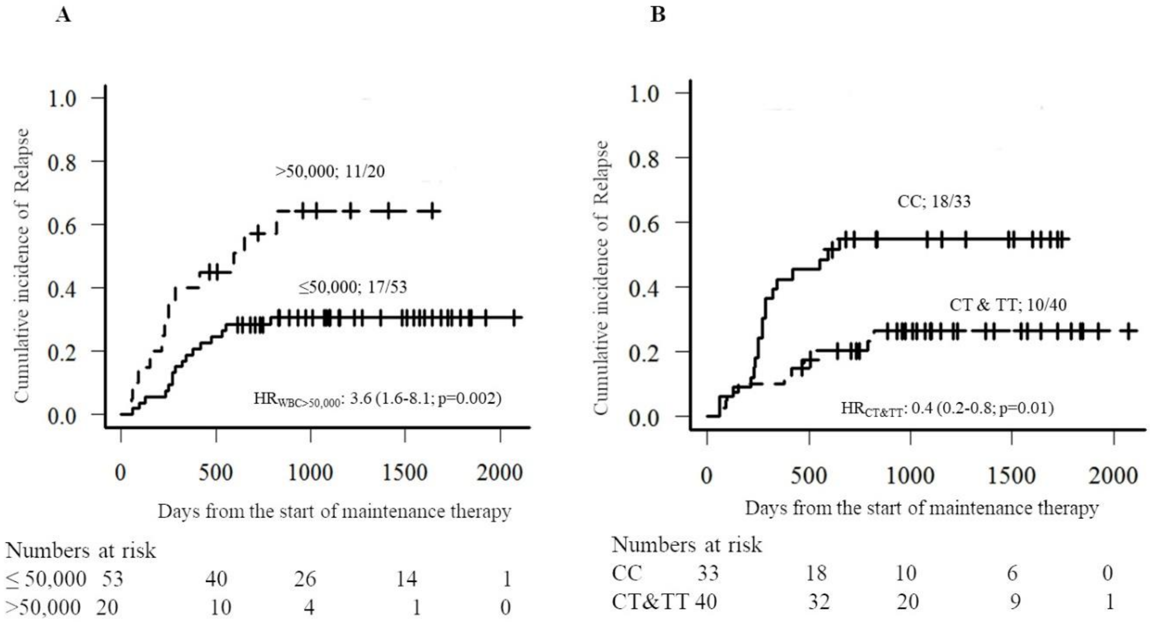

Figure S3: Association of incidence of relapse with that of; (A) WBC count at the time of diagnosis (n=73); (B) *ABCB1* or *MDR1* variant c.3435 T>C (n=73). Numbers at risk in each group at each time point indicates the number of individuals without the event. The number of events / total numbers in each group, p values and hazards ratios for cumulative incidences are presented on the plots (multivariate, only *ABCB1* explained relapse incidence, due to limited numbers other factors were eliminated from the model). This analyses represent exploratory findings, as the p values after multiple testing is not significant, possibly due to low number of the study subjects.

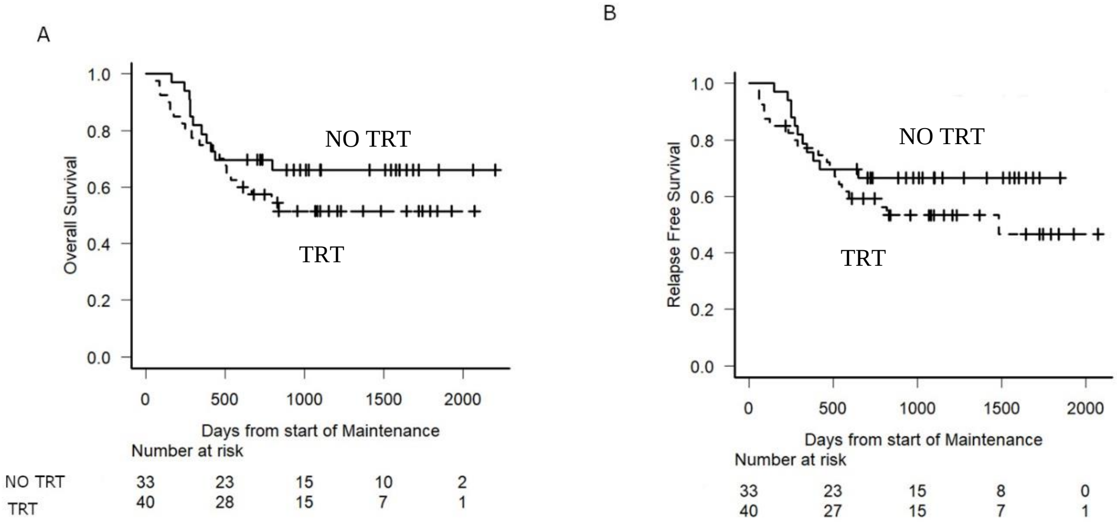

Figure S4: Association of Overall Survival (A) and Relapse free survival (B) in relation to early treatment related haematological toxicity sever grades 3-4. Numbers at risk in each group at each time point indicates the number of individuals without the event. Censored patients are marked on the plots. This analyses represent exploratory findings due to low number of study patients, p values are not significant.
